# Supplementary material for: Ventral pallidum encodes relative reward value earlier and more robustly than nucleus accumbens
Source: Nat Commun. 2018 Oct 19;9:4350. doi: 10.1038/s41467-018-06849-z (PMC6195583; doi:10.1038/s41467-018-06849-z)
Supplement: Supplementary file 1 — Supplementary Information. [file 41467_2018_6849_MOESM1_ESM.pdf]

# Ventral pallidum encodes relative reward value earlier and more robustly than nucleus accumbens

David Ottenheimer, Jocelyn M. Richard, Patricia H. Janak

Correspondence: david.ottenheimer@jhu.edu, patricia.janak@jhu.edu

**Supplementary figures:**

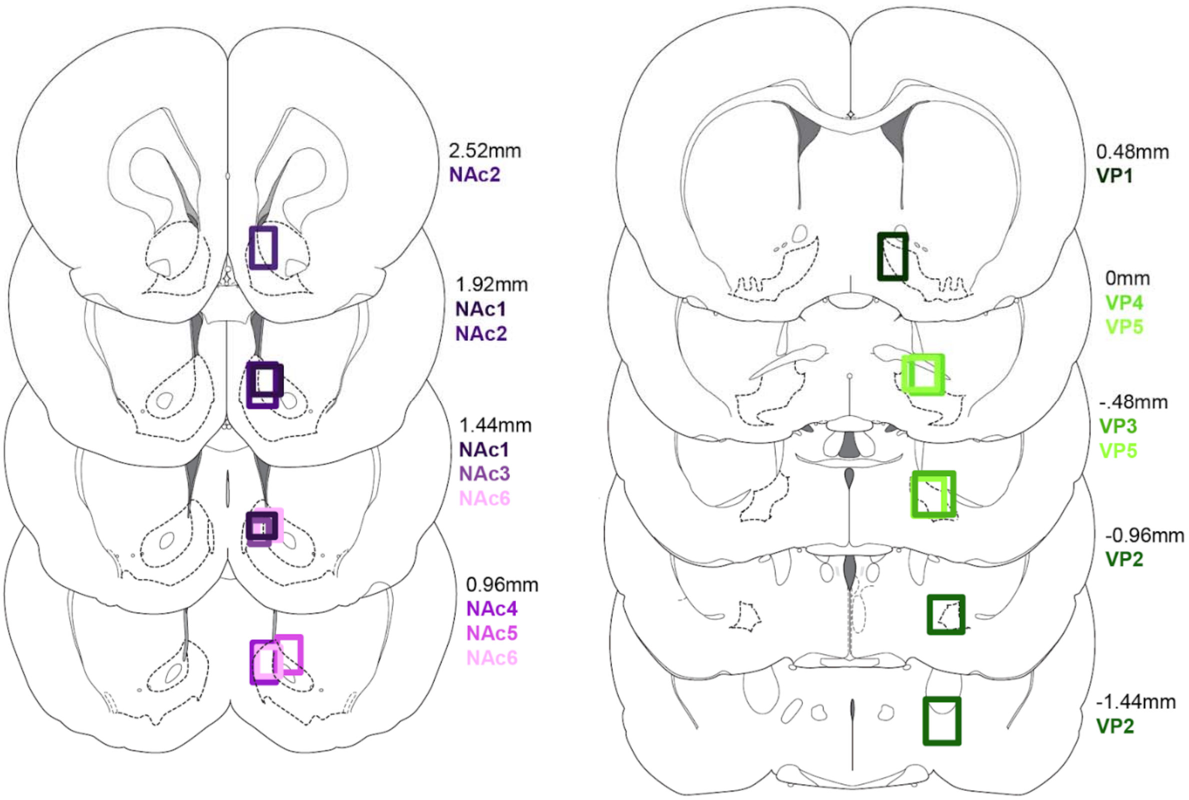


**Supplementary Figure 1. Recording locations.**
Dashed lines demarcate nucleus accumbens shell and core and ventral pallidum. Placements are color coded by rat. The posterior portion of VP2's placement included extended amygdala.

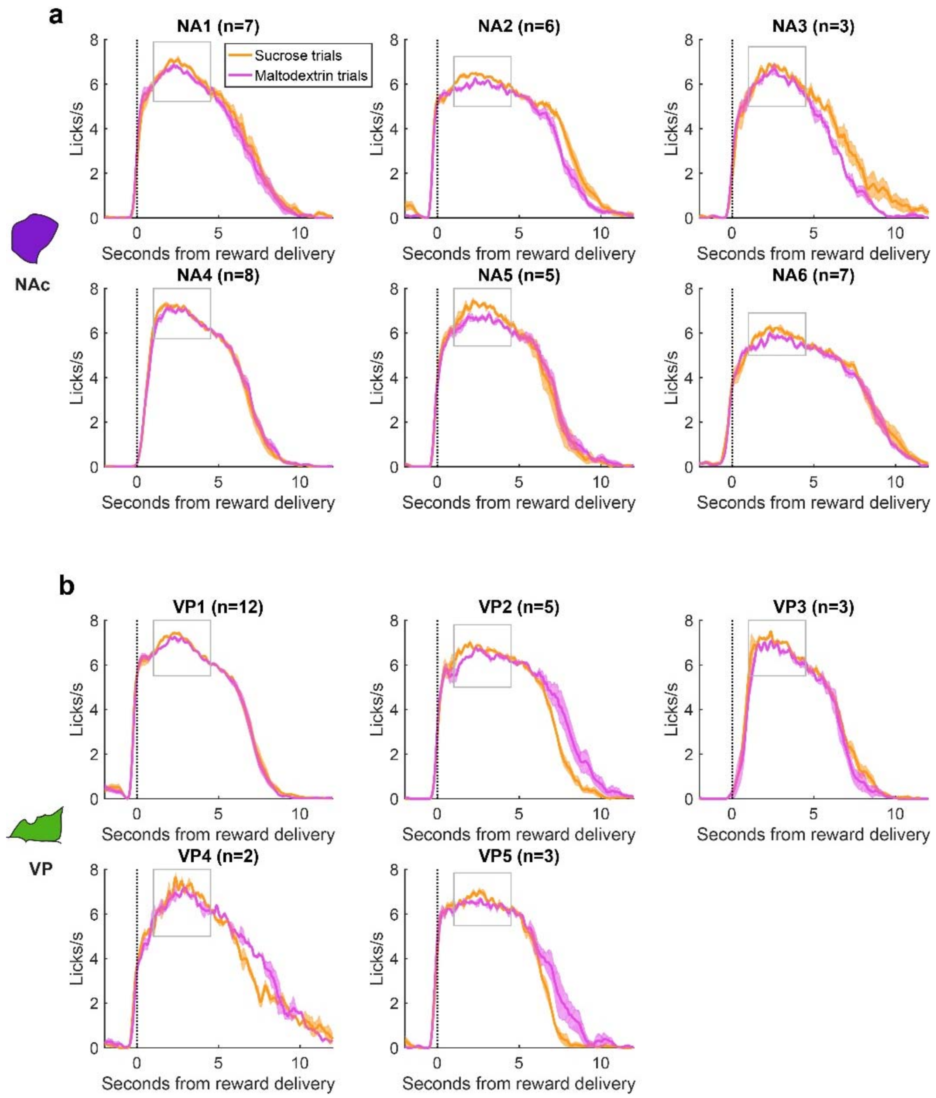


**Supplementary Figure 2. Licking behavior in each rat.**
(**a**) Mean lick rate for each individual NAc rat on sucrose (orange) and maltodextrin (pink) trials (n is each included session). Gray box indicates time of interest (1-4.5s following reward delivery) when licking is consistently greater for sucrose than for maltodextrin. (**b**) As in (a), for VP rats.

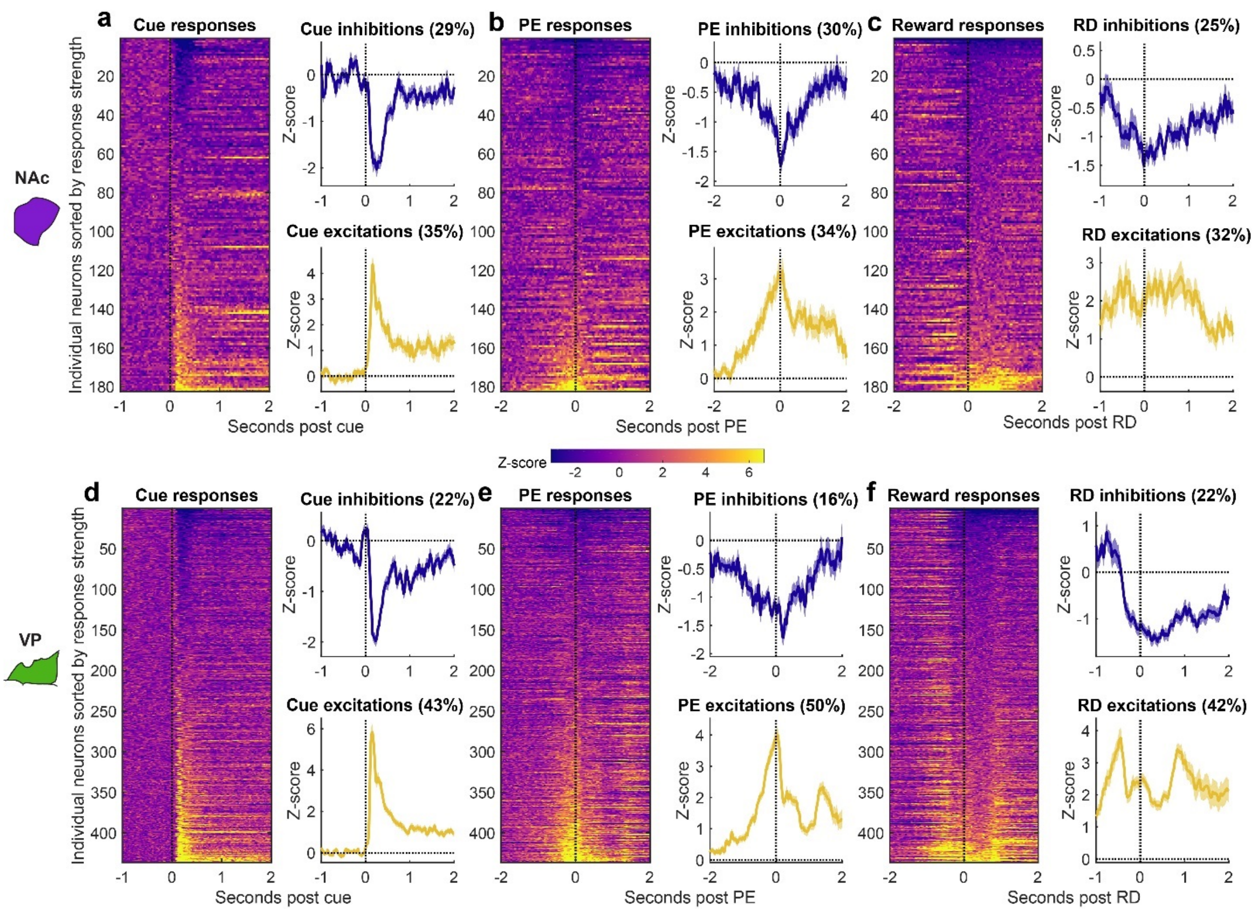


**Supplementary Figure 3. Neural responses to reward-related events.**
(**a**) Left: All NAc neurons sorted by firing rate 500ms following cue onset. Right: Neurons significantly inhibited (blue) and excited (yellow) by cue onset. Shading is SEM. (**b**) As in (a), for the 1000ms centered on port entry (PE). (**c**) As in (a), for the 1000ms following reward delivery (RD). (**d-f**) As in (a-c), for neurons from VP.

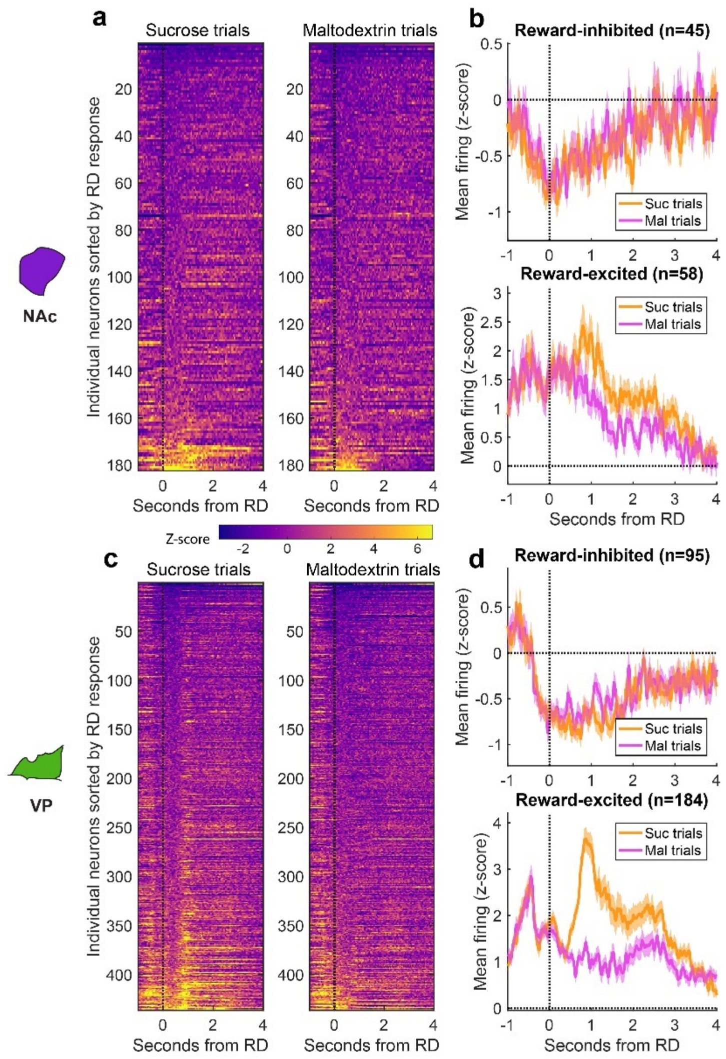


**Supplementary Figure 4. Average reward responses on sucrose and maltodextrin trials.** (**a**) Individual neurons' normalized responses on sucrose and maltodextrin trials. Neurons are sorted the same as Supplementary Figure 3c. (**b**) Average activity of significantly inhibited and excited neurons (as in Supplementary Figure 3c) separated into sucrose and maltodextrin trials. Shading is SEM. (**c,d**) Same as (a) and (b) for VP neurons.

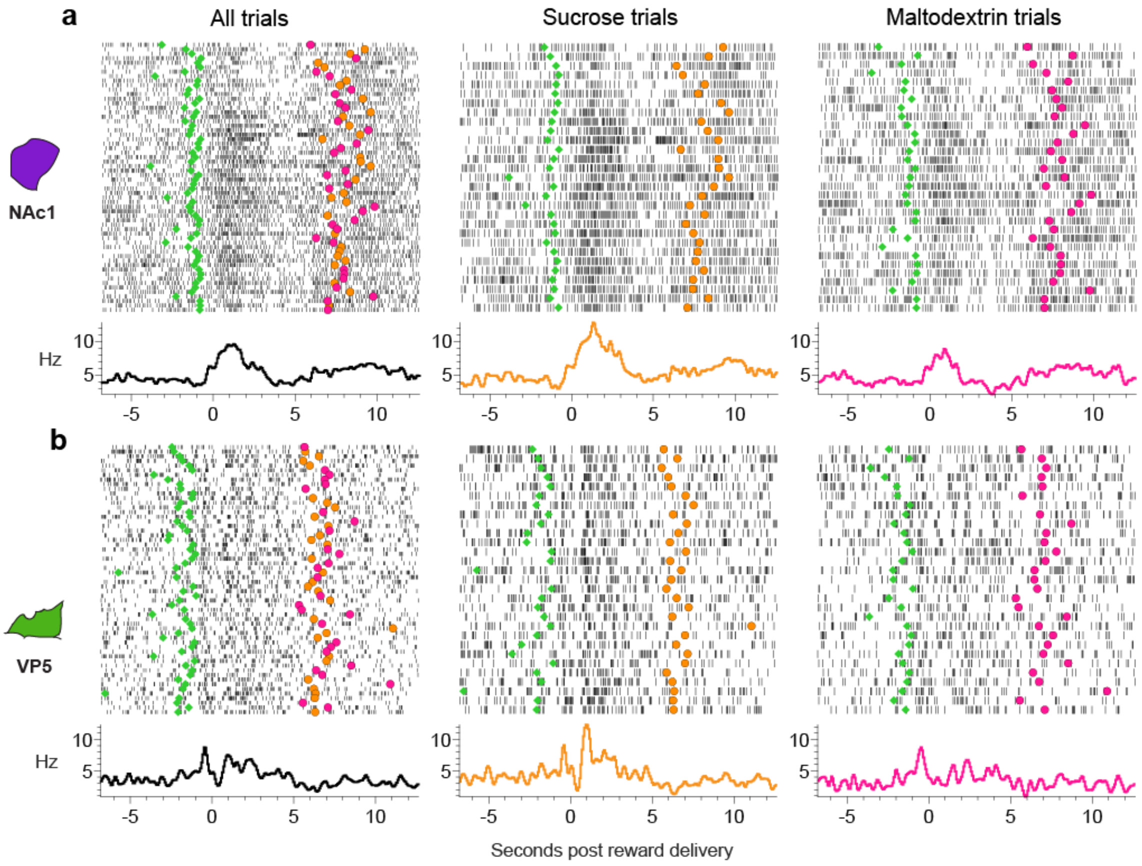


**Supplementary Figure 5. Example reward-selective neurons.**
(**a**) Perievent raster (top) and histogram (bottom) of a reward-selective neuron from NAc1 aligned to reward delivery for all (left), sucrose (center) and maltodextrin (right) trials. Green diamond is cue, orange circle is final sucrose lick, pink circle is final maltodextrin lick. Perievent histogram constructed with 75ms bins and smoothed with a Gaussian filter over 3 bins. (**b**) As in (a), for a reward-selective neuron from VP5.

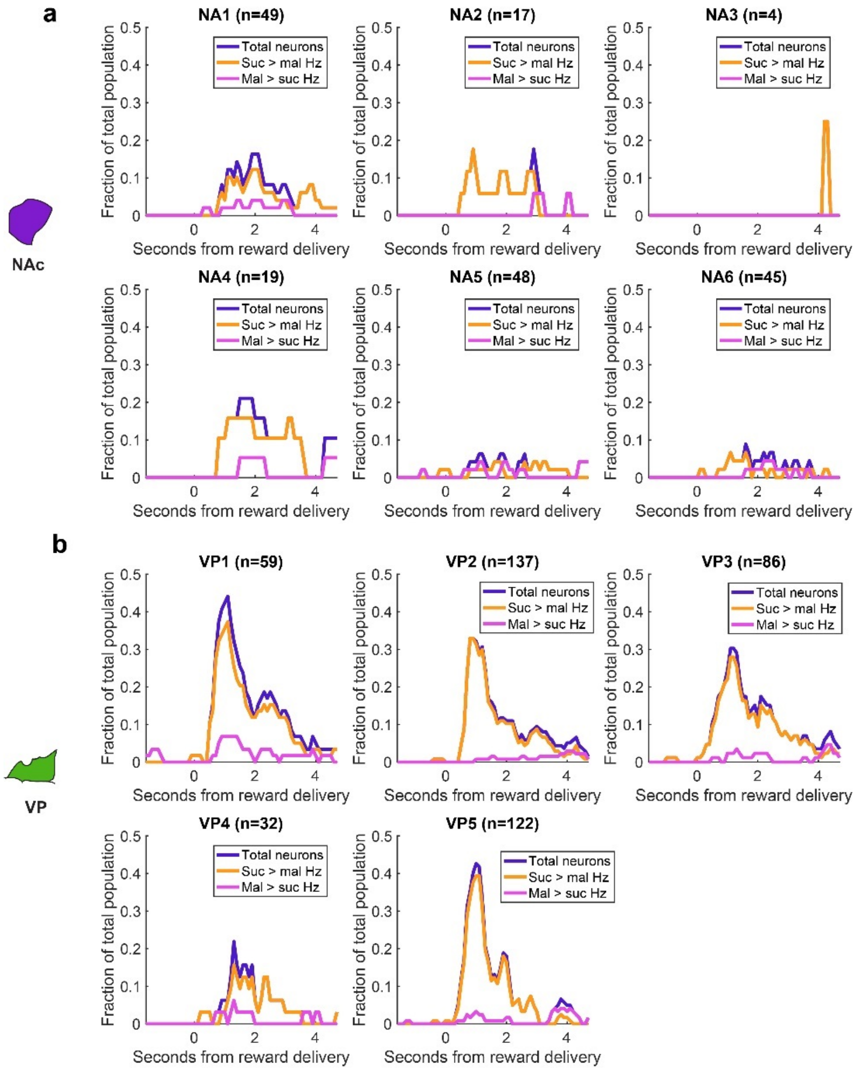


**Supplementary Figure 6. Reward-selective neurons in each rat.**
(**a**) Histogram of the fraction of neurons in each NAc rat that meet criteria for reward selectivity in overlapping 600ms bins (advanced by 100ms) (see Figure 2a). Plotted are the total fraction of reward-selective neurons (blue) and, of those, neurons with greater firing for sucrose (orange) and greater firing for maltodextrin (pink). (**b**) As in (a), for VP rats.

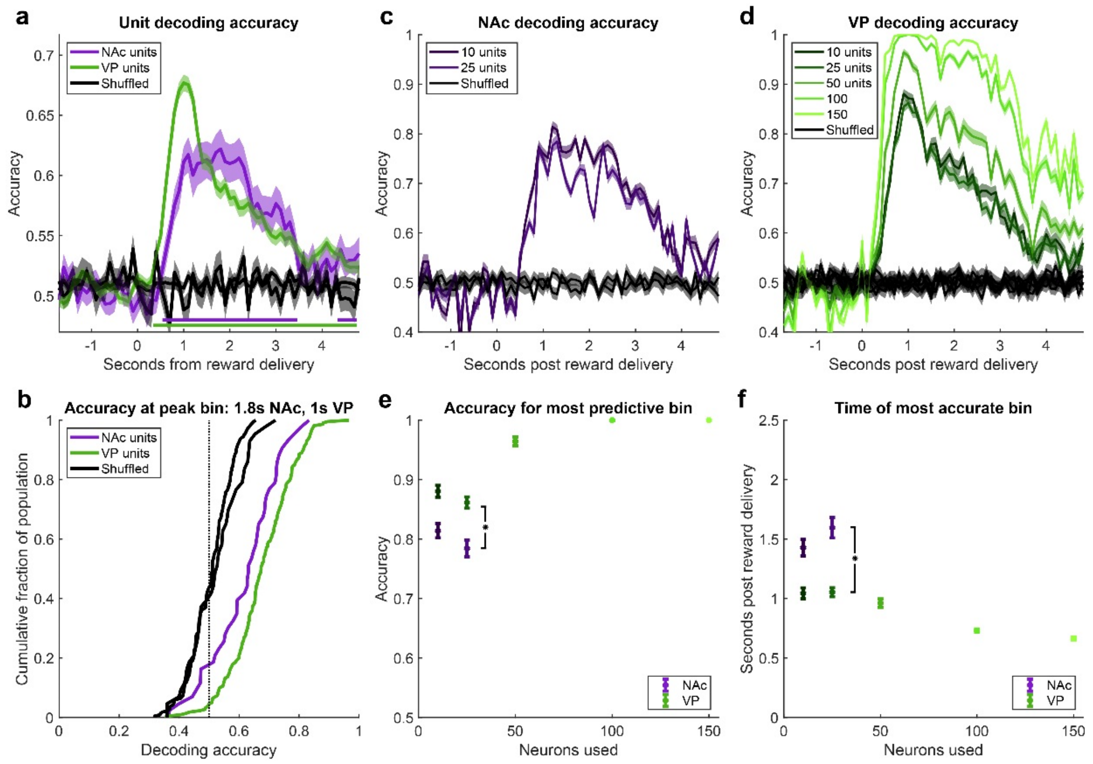


**Supplementary Figure 7. Decoding trial identity with only reward-selective neurons.** **(a)** Average cross-validated decoding accuracy relative to reward delivery time, determined using linear discriminant analysis models trained on spiking data of individual reward-selective neurons (as identified in Fig. 2) across 600ms overlapping bins. Decoding accuracy for NAc (purple), VP (green), and data with shuffled trial identity from each region (black). Shading is SEM. Purple (NAc) and green (VP) lines indicate consecutive bins where accuracy exceeds 99% confidence interval of corresponding shuffled data. **(b)** Cumulative distribution of accuracies in the bin with the greatest average accuracy in each region (centered at 1.6s in NAc and 1s in VP) and the corresponding shuffled data from that bin in each region. **(c)** Average cross-validated decoding accuracy relative to reward delivery time of linear discriminant analysis models trained on spiking data of 20 randomly selected groups of 10 or 25 neurons in NAc across 600ms overlapping bins and corresponding models trained on data with trial identity shuffled. Shading is SEM. **(d)** Same as (c) for VP pseudoensemble models of 10, 25, 50, 100, or 150 neurons. **(e)** Average accuracy of each replicate for the bin with peak accuracy for each pseudoensemble size in each region. Asterisk indicates significant main effect of region on accuracy for 10 and 25 neuron ensembles ($F(1,196) = 38.9$, $p = 2.7E-9$). **(f)** Average peak accuracy time post-reward for each replicate of each pseudoensemble size in each region. Asterisk indicates significant main effect of region on time of peak accuracy for 10 and 25 neuron ensembles ($F(1,196) = 54.8$, $p = 3.8E-12$).

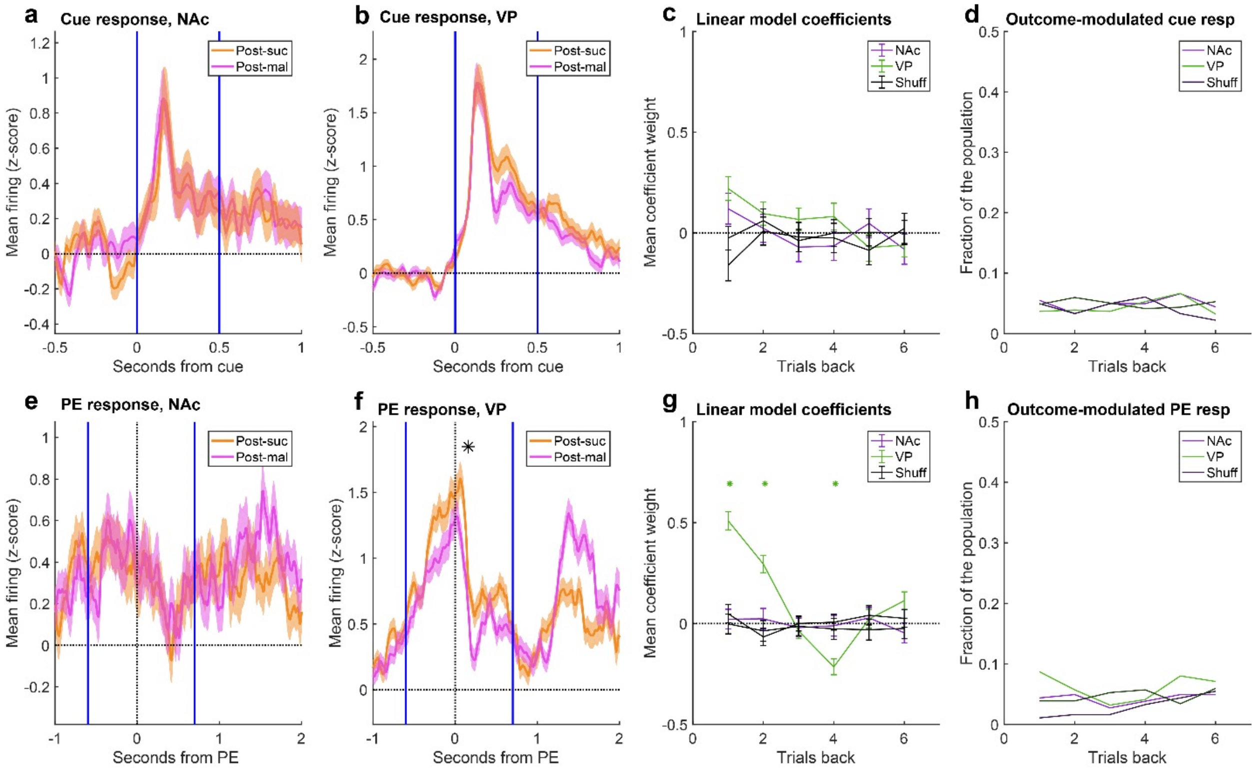


**Supplementary Figure 8. Impact of previous reward outcomes on cue- and port entry-evoked firing.**

**(a,b)** Normalized cue-evoked activity of all neurons in NAc (n = 182) (a) and VP (n = 436) (b) on trials with sucrose (orange) or maltodextrin (pink) as the most recent reward outcome. Blue lines indicate epoch selected for analysis of the impact of previous outcome in (c,d) (0-0.5s from cue onset). **(c)** Mean coefficient weights for the impact of the previous 6 trials on normalized firing rate for each neuron in NAc (purple), VP (green), and corresponding data for each neuron with the outcomes shuffled (black) for each region. Error bars are SEM. **(d)** Proportion of the neural populations in VP (green), NAc (purple), and corresponding shuffled neurons (black) with significant coefficients for each of the relative trials. **(e,f)** As in (a,b) for port entry-evoked activity. Blue lines indicate epoch selected for analysis of the impact of previous outcome in (g,h) (-0.6 - 0.7s from port entry). Asterisk indicates significant effect of previous reward on port entry-evoked activity in VP ($F(1,862) = 16.7$, $p = 0.00048$ **(g)** As in (c), for port entry activity. Asterisks are $p < 0.05$ for Tukey tests comparing VP coefficients to shuffled data, corrected for multiple comparisons. **(h)** As in (d), for port entry models.
